# Affordable Microfluidic Bead-Sorting Platform for Automated Selection of Porous Particles Functionalized with Bioactive Compounds

**DOI:** 10.1038/s41598-019-42869-5

**Published:** 2019-05-10

**Authors:** Sahand Saberi-Bosari, Mohammad Omary, Ashton Lavoie, Raphael Prodromou, Kevin Day, Stefano Menegatti, Adriana San-Miguel

**Affiliations:** 1 Department of Chemical and Biomolecular Engineering, NC State University, Raleigh, NC 27695 USA; 2 Biomanufacturing Training and Education Center (BTEC), NC State University, Raleigh, NC 27695 USA

**Keywords:** Chemical engineering, Lab-on-a-chip

## Abstract

The ability to rapidly and accurately evaluate bioactive compounds immobilized on porous particles is crucial in the discovery of drugs, diagnostic reagents, ligands, and catalysts. Existing options for solid phase screening of bioactive compounds, while highly effective and well established, can be cost-prohibitive for proof-of-concept and early stage work, limiting its applicability and flexibility in new research areas. Here, we present a low-cost microfluidics-based platform enabling automated screening of small porous beads from solid-phase peptide libraries with high sensitivity and specificity, to identify leads with high binding affinity for a biological target. The integration of unbiased computer assisted image processing and analysis tools, provided the platform with the flexibility of sorting through beads with distinct fluorescence patterns. The customized design of the microfluidic device helped with handling beads with different diameters (~100–300 µm). As a microfluidic device, this portable novel platform can be integrated with a variety of analytical instruments to perform screening. In this study, the system utilizes fluorescence microscopy and unsupervised image analysis, and can operate at a sorting speed of up to 125 beads/hr (~3.5 times faster than a trained operator) providing >90% yield and >90% bead sorting accuracy. Notably, the device has proven successful in screening a model solid-phase peptide library by showing the ability to select beads carrying peptides binding a target protein (human IgG).

## Introduction

The ability to rapidly identify small synthetic molecules capable of binding biological targets (*e.g*., single proteins, viruses, cells) with high affinity and selectivity is key to the development of novel drugs, diagnostic reagents, biosensing moieties, and ligands for the purification of biotherapeutics^1–7^. The introduction of solid-phase combinatorial libraries of synthetic compounds has resulted in an exponential growth of bioactive compounds. Peptides and peptide mimetics are among the most utilized class of molecules, owing to their innate affinity for biological targets^8–10^ and the rich variety of strategies and amino acid building blocks available for peptide synthesis^11–14^. Bioinformatic tools have also emerged that guide the design of peptide libraries in terms of peptide length, structure, and amino acid composition, based on molecular-level information on the biomolecular target and putative binding sites on its surface^15,16^. Together with library design, the technology for library screening is crucial to identify peptide sequences with high binding affinity. With the growth of basic research utilizing protein-binding ligands and bioactive compounds, a number of high-throughput systems for the screening of combinatorial libraries have been developed and made commercially available. This instrumentation, however, is often cost-prohibitive to most academic groups^17^, and alternative cost-effective solutions are highly sought after.

Solid-phase peptide libraries comprise a large number (from thousands to millions) of small porous beads, wherein every bead carries multiple copies of a unique peptide sequence. Throughout the screening process, beads are *(i)* incubated with a labeled target, often in presence of other impurities, *(ii)* sorted using a detector that recognizes beads that have captured the labeled target, and finally *(iii)* analyzed to identify the peptide sequence they carry^5,6,18–20^. Commercial beads feature a polydispersed distribution of sub-millimeter diameters, and capture an amount of labeled target that likely depends not only on the binding affinity of the peptide they carry, but also on their particle diameter (~100 µm-300 µm) and pore size distribution. This inherent variability makes library screening and selection of candidate beads extremely labor intensive and reliant on the operator’s ability and subjective visual inspection. To streamline solid-phase screening and ensure rigorous peptide selection, fluorescence-activated cell sorting (FACS) has been previously used for screening peptide libraries^21^. However, when using large beads as solid substrates (~100–300 µm), sorting using FACS is not feasible. Instruments that address the size incompatibility issue from FACS screening, such as the Union Biometrica COPAS Flow Pilot system for library screening, have been made commercially available^18,19,22^. Hintersteiner *et al*. also developed a confocal nanoscanning and bead picking (CONA) platform for high-throughput screening of one-bead one compound libraries^23–25^. Their cost, accessibility, flexibility, and manufacturability, however, renders them prohibitive for academic research groups. Hintersteiner and coworkers cleverly sought to address this need with low-cost instrumentation for rapid picking from a monolayer of beads based on imaging by wide-field fluorescence microscopy^17^; this technology, however, does not allow for automated sorting and requires operator-directed selection of beads.

**Figure 1:**
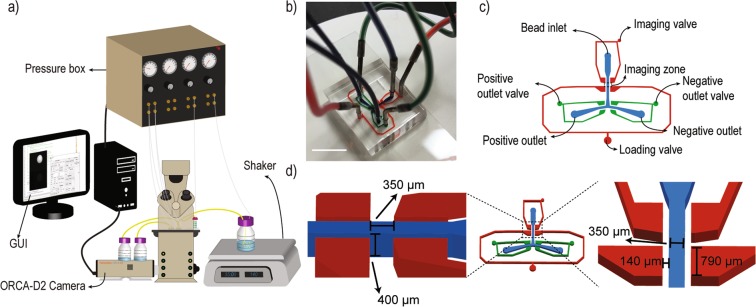
Microfluidic device for automated screening of bioactive compounds. (**a**) Schematic of experimental setup. (**b**) Photograph of the microfluidic platform. (**c**) Device schematic. Beads enter the device through the bead inlet and are trapped in the imaging zone by on-chip valves. Beads are then directed to positive or negative outlets depending on their fluorescence profile. (**d**) The width and length of imaging zone is 350 µm while the height is 400 µm. The PDMS membrane separating flow and valve channels is 140 µm. Scale bar is 1 cm.

Microfluidic platforms have previously been used to sort particles, cells and droplets^26–29^. We therefore sought to develop a low-cost, accessible, and portable platform for inexpensive, automated sorting of library beads featuring equipment that is either present in academic laboratories or can be inexpensively fabricated, and yet that provides rigorous selection of peptide ligands with high affinity and selectivity for biological targets (Fig. 1a–c). This system enables (1) simultaneous positive and negative selection by orthogonal labeling to isolate peptide binders with high affinity for a target and low-to-no binding for biological competitors, (2) rapid library screening with a rate of up to 100–150 beads per hour, (3) handling beads with different diameters (~100–300 µm), (4) ability to integrate library screening with external stimuli to select peptides that feature stimuli-controlled protein binding, and (5) identification of diverse targets of varying sizes by providing image-processing based spatial binding distribution analysis. Our platform is adaptable to different detection and sorting modes, providing the flexibility to screen for a variety of targets. To this end, our system comprises a microfluidic bead-sorting chamber, a dual wavelength fluorescent microscope, and customized software for real-time bead monitoring, image processing, and sorting. The microfluidic bead-sorting chamber is manufactured by photo-lithography followed by soft-lithography^30^. The software, based on a MATLAB Graphical User Interface, enables rapid (<0.75 sec) acquisition of fluorescent bead images, followed by image-processing based analysis and sorting (to either the selection receptacle or to waste). Notably, the quantitative image analysis algorithm takes into account both intensity and radial distribution of fluorescence, which combined improves the sensitivity and specificity of the platform in selecting high-affinity peptides by reducing the risk of false positives and negatives. To ensure identification of peptides with high binding selectivity, the algorithm enables orthogonal dual-color sorting by selecting the beads that only capture the fluorescently-labeled target while excluding beads that carry competitor proteins labeled with a different color. Finally, the device can be operated in either bulk or single bead separation mode. In bulk separation mode all positive beads are directed to a common flask, whereas in single bead separation mode each positive bead is individually directed to a single well in a 96 well-plate. In this work, we validated the integrated bead-sorting device and code using a dual fluorescence library mimetic. This comprises a combination of ChemMatrix beads labeled either by covalent conjugation of fluorescent dyes (Texas Red and Fluorescein) or by adsorption of fluorescently-labeled, streptavidin-conjugated proteins of different molecular weight to biotinylated beads resulting in either a homogeneous or halo-like radial color distribution, respectively. The fluorescence intensity and radial pattern observed in each class of beads were used as metric to inform sorting parameters. Beads with different fluorescence patterns (homogenous or halo-like) were analyzed to quantify the accuracy of bead-sorting protocol. Our results indicate that the device was capable of sorting beads with a detection yield and accuracy of ~92% and 94% respectively. It should be noted that the system performs all sorting and quantitative image analysis without any user supervision, thereby enabling a fully automated library screening process. It is worth mentioning that high sensitivity and specificity were achieved while microscopy was performed in a non-confocal setup demonstrating the robustness of algorithm in handling images with suboptimal quality. The flexibility of quantitative image analysis algorithm in handling images with various qualities, enables the set up to be used with different instruments. In this work, we designed and fabricated an automated, low-cost, accessible, and portable microfluidic platform capable of sorting targets with various fluorescence patterns with high precision and accuracy.

## Results and Discussion

Peptides and peptide mimetics with high affinity and selectivity for biological targets will play a critical role in next generation medicinal chemistry, diagnostics, and downstream biomanufacturing technology^2,3,31–34^. Streamlining their identification requires affordable, automated devices capable of sorting solid-phase peptide library beads with accurate and rigorous orthogonal selection logics. To meet these needs, we have developed a low-cost accessible automated microfluidic platform to handle, capture, image, analyze, and sort beads based on fluorescence patterns that directly translate into the binding affinity of the peptides carried by the selected library beads. We applied this technology for the isolation of positive (*i.e*., protein binding) beads from a library mimetic consisting of a mixture of labeled ChemMatrix beads with different fluorescent color, intensity, and radial distribution.

### Diverse fluorescence patterns for protein binding detection

**Figure 2:**
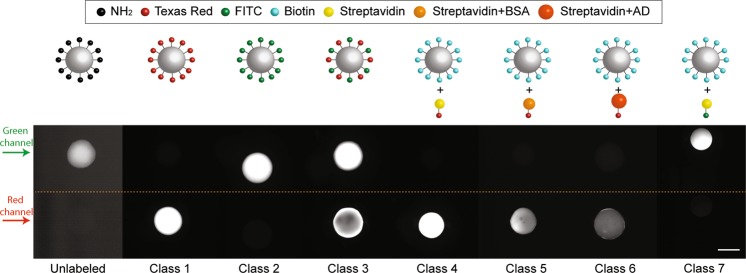
Various classes of beads with different fluorescence profiles. Beads in classes 1–3 exhibit homogeneous fluorescence patterns in green, red, and green/red. Class 4 and 7 were prepared by adsorbing red and green Streptavidin onto biotin beads producing broad halo pattern in red and green channel respectively. Classes 5–6 were prepared by adsorbing red-Streptavidin-BSA and red-Streptavidin-AD onto biotin beads producing medium and narrow halo pattern respectively. The scale bar is 200 µm. *Contrast has been modified in the Unlabeled class image for visibility*.

In prior studies^5,6,19,35^, PEG-based ChemMatrix resin has been successfully utilized as a substrate for the synthesis and screening of solid-phase peptide libraries against target proteins for the selection of peptide ligands. In this context, however, the small pore diameter of ChemMatrix resin has been shown to limit protein diffusion through the bead, resulting in a protein-rich corona, or “halo” whose width decreases with the molecular weight of the protein^18^. When fluorescently labeled targets are utilized, this translates in a halo-like fluorescence pattern; in theory, smaller proteins produce a diffuse halo, while larger ones produced a narrow halo. To replicate this effect, a number of fluorescent bead classes were prepared, either by conjugating different amine-reactive fluorophores (*i.e*., Texas Red NHS and FITC) onto aminomethyl ChemMatrix beads or by adsorbing fluorescently labeled streptavidin or streptavidin-conjugated proteins of different molecular weight onto biotin-ChemMatrix beads (Fig. 2). Specifically, seven classes of beads were prepared, namely: Class 1, homogeneous only-red fluorescence, prepared by conjugating amine-reactive Texas Red to aminomethyl ChemMatrix beads; Class 2, homogeneous green-only fluorescence, prepared by reacting Fluorescein isothiocyanate (FITC) with aminomethyl ChemMatrix beads; Class 3, homogeneous dual red-green fluorescence, prepared using both Texas Red and Fluorescein; Class 4, broad halo only-red fluorescence, prepared by adsorbing red-Streptavidin (MW ~ 56 kDa) onto biotin-ChemMatrix beads; Class 5, medium halo only-red fluorescence, prepared by adsorbing Texas Red-labeled Streptavidin-BSA conjugates (MW ~ 118 kDa) onto biotin-ChemMatrix beads; Class 6, narrow halo only-red fluorescence, prepared by adsorbing Texas Red-labeled Streptavidin-AD (MW ~ 202 kDa) conjugates onto biotin-ChemMatrix beads; and Class 7, broad halo only-green fluorescence, prepared by adsorbing green-Streptavidin (MW ~ 56 kDa) onto biotin-ChemMatrix beads.

### Flexible automated detection, analysis, and sorting of beads

To enable automated bead selection and sorting, we developed customized algorithms to detect the presence of a bead, to extract image-based descriptive metrics required for classification, and to sort the bead according to the criteria provided by the operator into a positive line to the collection receptacle or a negative line to be discarded. Bead selection was based on (i) radial distribution of fluorescence, (ii) fluorescence intensity, and (iii) difference of color 1 *vs*. color 2 intensities. Radial distribution of fluorescence depends on the diffusion of the target biomolecule through the bead, and thus on its hydrodynamic radius; “halo”-like radial distribution can be utilized as a criterion of choice when screening against large protein targets, so that beads showing a homogeneous fluorescence distribution are discarded as false positives. Fluorescence intensity correlates to the ability of the peptides carried by the bead to effectively capture the target biomolecule; thus, selection of beads carrying high intensity promotes the selection of peptides with high binding strength. Finally, the difference of intensities in two colors, is utilized for selection of ligands with high binding selectivity. Library selection is performed in “competitive conditions”, that is, by co-incubating the labeled target protein with a myriad of other protein impurities communally labeled orthogonally to the target (*e.g*., using fluorescence dyes with non-overlapping emission wavelengths); accordingly, the selection of beads carrying only the desired label ensures capture specificity. To demonstrate the full potential of our technology, we sorted the beads from the library mimetic according to various fluorescence patterns and intensities, showing that the device is capable of unsupervised bead sorting with high accuracy in response to criteria set by the user.

**Figure 3:**
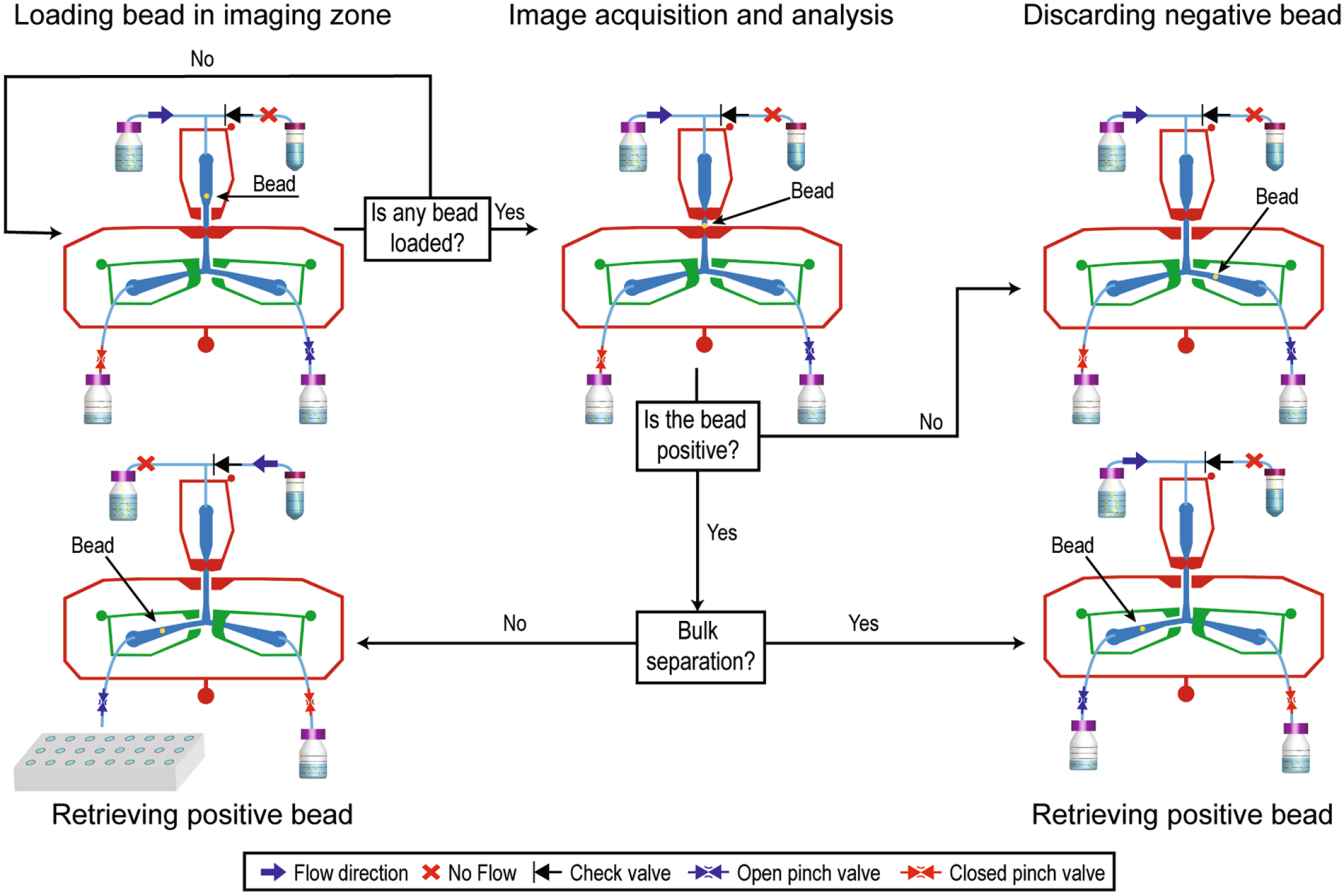
Screening process flowchart. Beads are flown through the device while the loading valve is closed and the imaging valve open. Once a bead is detected in the imaging zone, the imaging valve closes trapping the bead. If the bead is assessed to be negative, the loading valve will open up and allow the bead to flow through the negative outlet. If the bead is assessed to be positive, the negative outlet valve closes and the positive outlet valve opens while the loading valve opens and allows the bead to flow through the positive outlet.

Screening the peptide library using the microfluidic platform comprises a series of tasks performed in a loop. Throughout the process, on/off-chip valves are utilized to trap single beads in the imaging zone and sort them as positive or negative based on the selection criteria. The first step involves loading an individual bead to the imaging zone (Fig. 3, Top left); in this step, the beads are withdrawn from the suspension and flown through the device, while keeping the loading and positive outlets valves closed, and the negative outlet and imaging valve open. Importantly, the valves used in this system are only closed partially, thus trapping beads while allowing fluid to flow through closed valves. As the fluid flows through the imaging zone, the algorithm is constantly acquiring and analyzing frames from the camera to detect the presence of a bead. Detecting a bead triggers the second step, where the imaging, loading and positive outlet valves are closed, and the bead is retained in the imaging zone (Fig. 3, Top middle). A second image is then acquired to ensure the presence of a bead in the imaging zone. This image is fed to an image processing algorithm that segments the bead and extracts various descriptive metrics. The system then determines whether the bead is positive by comparing these metrics with the thresholds input by the operator. If the bead is assessed as negative, the loading valve opens and allows the bead to be expelled through negative outlet, while flow through positive outlet is stopped by both on and off chip valves (Fig. 3, Top right). Off-chip pinch valves are used in both positive and negative outlets to ensure the flow is completely stopped. As mentioned earlier, collection of positive beads can be performed in two different modes, either by transferring individual beads to single wells in a 96 well plate or by collecting them in bulk in a common flask (Fig. 3, Bottom right and left). When operating in bulk collection mode, once a positive bead is detected, the negative outlet valves (on and off-chip) are closed while the positive outlet valves and the loading valve open, allowing the bead to flow toward the flask. The incoming fluid from the reservoir containing the bead suspension is sufficient to direct the bead to the collection flask. When operating in single bead collection mode, once a positive bead is detected, the system is programmed to pause the operation and ask the operator for permission to continue. As the positive outlet tube is placed in the designated well, upon receiving permission to continue by the operator, the system opens the positive outlets and the loading valves, allowing the bead to travel to the collection well with the flow provided by the flush stream. With this set up, were able to perform sorting at a speed of up to 125 beads/hr (~3.5 times faster than a trained operator) (Supplemental Fig. 2).

### Sorting and detection validation

Several tests were performed to evaluate the bead sorting accuracy and precision of the system using fluorescently labeled beads of Class 1–7. Library mimetics depicting different screening scenarios were prepared by mixing beads of one class, considered as positive, with a combination of beads from other classes. In every experiment, a different library mimetic was suspended in PBS at a density of ~2 beads/mL and maintained under gentle agitation to prevent aggregation. Beads were fed at a rate of ~2.5 beads/min. The beads conforming to the set selection criteria were sorted as positive, and subsequently analyzed to calculate the yield and accuracy of the sorting process, respectively defined as ratio of positive beads collected *vs*. positive beads fed and ratio of positive beads collected *vs*. total beads collected in the positive flask.

### Sorting beads with homogenous fluorescence patterns

**Figure 4:**
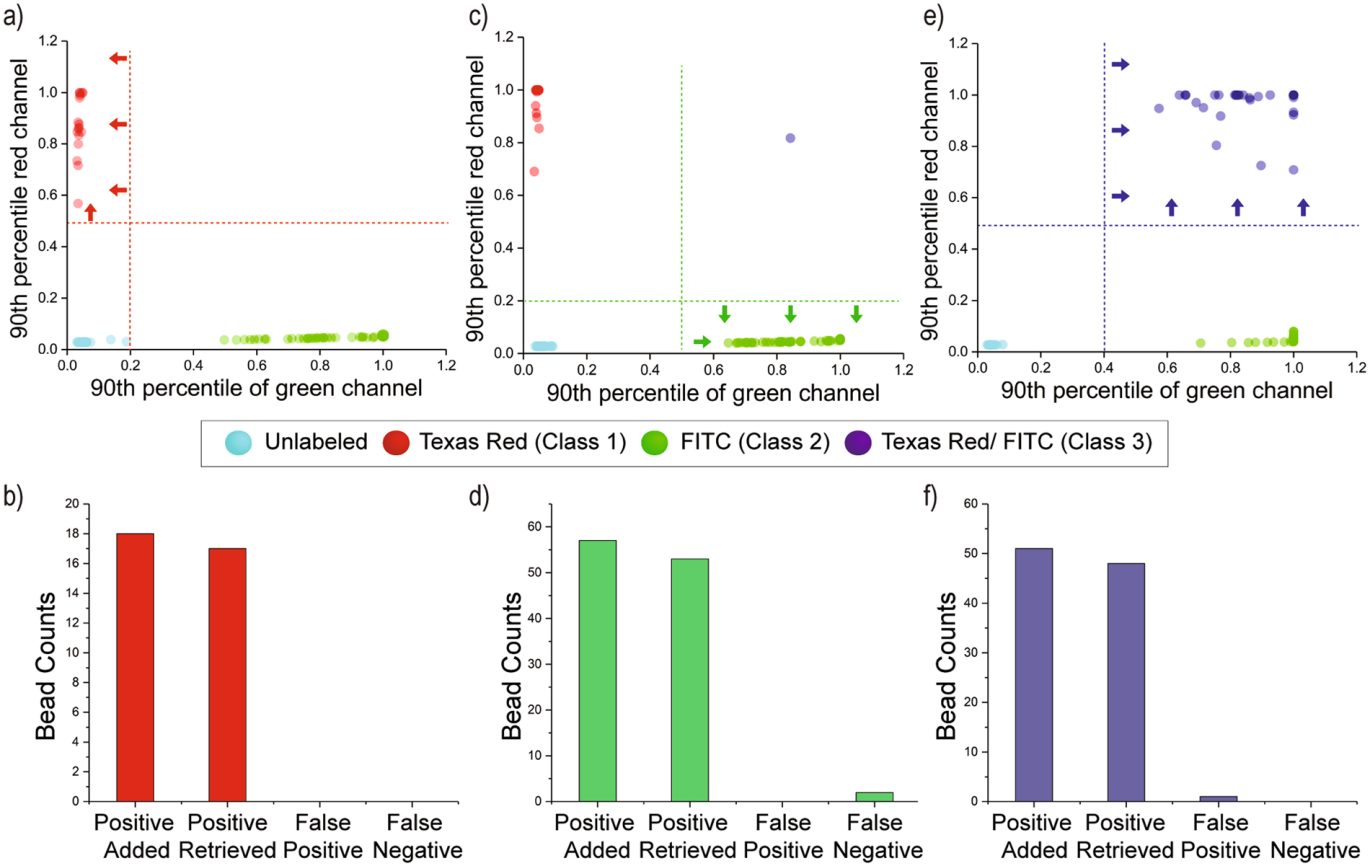
Detection and sorting of beads with homogeneous fluorescence patterns. (**a,c,e**) Dot plot of samples screened with the goal of sorting class 1,2, and 3 beads respectively. The values for decision hyperplanes (lines) were established based on preliminary data acquired for each class. The purple point in figure (c) occurred due to having class 1 and class 2 beads entering the imaging zone together. Both beads were sorted as negative. (**b,d,f**) Bar plots of the platform performance in sorting class 1, 2 and 3 beads respectively. “Positive added” is the known number of positive beads added to the flask. “Positive Retrieved” represents the true positives collected at the outlet. “False Positive” and “False Negative” are the beads sorted incorrectly.

We first processed beads with uniform high-intensity fluorescence patterns in either a single (red-only or green-only) or dual (red and green) color (Fig. 2). In the first test, beads with uniform red-only fluorescence (Class 1) were used as the positive set, and mixed with untreated ChemMatrix beads and green-only fluorescent beads (Class 2) as negatives. We established the positive selection criterion based on the 90^th^ percentile of the bead’s intensity in the red channel and the 90^th^ percentile of the bead’s intensity in the green channel. Beads exhibiting values above 0.5 in normalized red channel 90^th^ percentile and below 0.2 in normalized green channel 90^th^ percentile were considered positive (Fig. 4a,b). These threshold values were established based on preliminary images acquired from each bead class and analyzed to identify distinctive features. Using these thresholds, we were able to retrieve 17 out of the 18 positive beads initially present in the reservoir flask (~95% yield); while all 17 beads were confirmed as positive, indicating 100% accuracy.

The second test aimed to sort beads with green-only homogeneous fluorescence (Class 2) from a mixture of Class 2, Class 1, and unlabeled beads. For this test, beads with normalized 90^th^ percentile pixel intensity higher than 0.5 in the green channel and normalized 90^th^ percentile pixel intensity lower than 0.2 in red channel were considered positive (Fig. 4c,d). Using these thresholds, we retrieved 53 out of 57 initial positive beads (~93% yield); all 53 beads were confirmed as positive (100% accuracy). The third test aimed to isolate beads carrying dual red-green fluorescence (Class 3) from a mixture of Class 3, Class 2, and unlabeled beads. Beads were considered positive when the 90^th^ percentile of pixel intensity in the red and green channels was above 0.5 and 0.4, respectively (Fig. 4e,f). As a result, 48 positive beads and 1 false positive were retrieved out of 51 positive beads fed to the sorting device (~94% yield rate and ~98% accuracy).

### Sorting beads with “halo”-like fluorescence pattern

We then sought to evaluate the capability of this platform to sort beads with more complex fluorescence patterns. The homogeneous fluorescence distribution initially utilized is representative of library screening against small protein targets, which can easily diffuse through the pores of the library beads and be captured by the ligands displayed throughout the entire radius of the bead. In contrast, prior work by the Camperi group^18^ on screening ChemMatrix-based peptide libraries indicates that, due to diffusion limitations, larger target proteins effectively penetrate only the outer corona of the beads. This translates into a “halo”-like fluorescence pattern, potentially accompanied by fluorescing spots randomly distributed throughout the bead (Fig. 2). Accordingly, additional tests were designed to assess the sorting of beads with beads with halo-like fluorescence pattern.

We first sought to separate Class 6 (narrow halo) beads from a mixture of Class 6, Class 4 (broad halo), Class 7 (broad halo green-only), and unlabeled beads. To distinguish beads with subtler fluorescence patterns, a more complex set of parameters was defined. Specifically, to be accepted as positive, a bead ought to meet three criteria:

$${\alpha }_{er} > 0.15,{\alpha }_{er}-{\alpha }_{eg} > 0,{90}_{eg}^{th} < 0.1$$

$${\alpha }_{eg}=\frac{{M}_{eg}-{90}_{eg}^{th}}{{M}_{eg}}$$

$${\alpha }_{er}=\frac{{M}_{er}-{90}_{er}^{th}}{{M}_{er}}$$

$${90}_{eg}^{th}={90}^{th}\,{\rm{Percentile}}\,{\rm{pixel}}\,{\rm{intensity}}\,{\rm{of}}\,{\rm{entire}}\,{\rm{bead}}\,{\rm{in}}\,{\rm{green}}\,{\rm{channel}}$$

$${90}_{er}^{th}={90}^{th}\,{\rm{Percentile}}\,{\rm{pixel}}\,{\rm{intensity}}\,{\rm{of}}\,{\rm{entire}}\,{\rm{bead}}\,{\rm{in}}\,{\rm{red}}\,{\rm{channel}}$$

$${M}_{eg}={\rm{Maximum}}\,{\rm{pixel}}\,{\rm{intensity}}\,{\rm{of}}\,{\rm{entire}}\,{\rm{bead}}\,{\rm{in}}\,{\rm{green}}$$

$${M}_{er}=\,{\rm{Maximum}}\,{\rm{pixel}}\,{\rm{intensity}}\,{\rm{of}}\,{\rm{entire}}\,{\rm{bead}}\,{\rm{in}}\,{\rm{red}}$$

where α_er_ and α_eg_ measure the fluorescence homogeneity through the bead. Smaller α_er_ and α_eg_ numbers indicate homogenous distribution of fluorescence within the bead, whereas larger values of α_er_ and α_eg_ indicate a wider range between the maximum pixel value and the 90^th^ percentile. These threshold values were chosen based on preliminary analyses conducted on images acquired from beads of Class 4, 5, 6, and 7.

**Figure 5:**
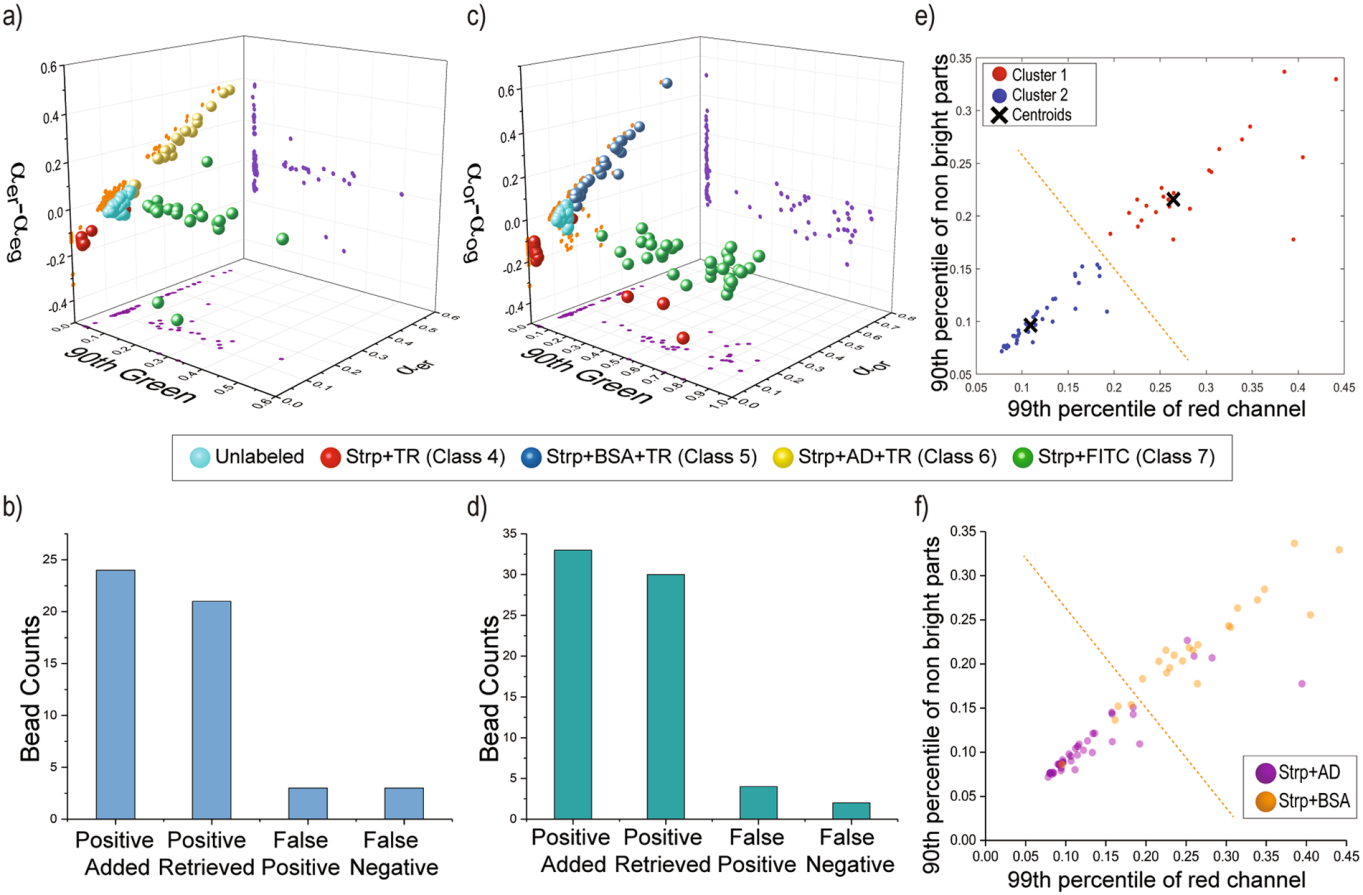
Capability of platform in sorting halo fluorescence patterns. (**a,c**) The 3D plot of samples screened to sort class 6 and 5 beads respectively. The values for decision hyperplanes (surfaces) were established based on preliminary data acquired for each class. (**b,d**) The bar plot of the platform performance in sorting class 6 and 5 beads respectively. “Positive Added” is the known number of positive beads added to the flask. “Positive Retrieved” represents the true positives collected at the outlet. “False Positive” and “False Negative” are the beads sorted incorrectly. (**e**) Unsupervised K-mean clustering of data extracted from images taken from class 5 and 6 beads. ~30 beads from each class were imaged and processed. (**f**) The ground truth labels for points used in the unsupervised K-mean clustering. These labeled data were used to calculate the accuracy of unsupervised clustering of data points acquired from class 5 and 6 beads.

As a result, 21 positive beads and 3 false positives were retrieved out of 24 positive beads fed to the sorting device (~87.5% yield). Considering that 3 beads sorted as false positive, we obtained a ~87.5% accuracy (Fig. 5a,b). The presence of false positive and false negative beads is imputed to the variability inherent to protein-peptide binding. In some instances, beads of Class 6 did not exhibit a halo-like pattern, likely due to heterogeneous pore size distribution through the bead. False positives and false negatives can also occur if two beads enter the imaging zone together, a result of aggregation or simple proximity in the flow. When two or more beads of different classes enter the imaging zone, the algorithm inevitably sorts them as either positive or negative, correspondingly resulting in false positive or false negative sorting. However, our data suggests the error caused by these phenomena is only a minor occurrence.

As a final experiment, we aimed to sort Class 5 beads (medium halo red-only fluorescence) from a mixture of Class 5, Class 4 (broad halo red-only), Class 7 (broad halo green-only), and unlabeled beads. Similar to the previous test, three criteria were defined to identify positive beads with non-uniform patterns:

$${\alpha }_{or} > 0.15,\,{\alpha }_{or}-{\alpha }_{og} > 0,\,{90}_{eg}^{th} < 0.1$$

$${\alpha }_{og}=\frac{{M}_{og}-{90}_{og}^{th}}{{M}_{og}}$$

$${\alpha }_{or}=\frac{{M}_{or}-{90}_{or}^{th}}{{M}_{or}}$$

$${90}_{eg}^{th}=\,{90}^{th}\,{\rm{Percentile}}\,{\rm{pixel}}\,{\rm{intensity}}\,{\rm{of}}\,{\rm{entire}}\,{\rm{bead}}\,{\rm{in}}\,{\rm{green}}\,{\rm{channel}}$$

$${90}_{og}^{th}=\,{90}^{th}\,{\rm{P}}{\rm{e}}{\rm{r}}{\rm{c}}{\rm{e}}{\rm{n}}{\rm{t}}{\rm{i}}{\rm{l}}{\rm{e}}\,{\rm{p}}{\rm{i}}{\rm{x}}{\rm{e}}{\rm{l}}\,{\rm{i}}{\rm{n}}{\rm{t}}{\rm{e}}{\rm{n}}{\rm{s}}{\rm{i}}{\rm{t}}{\rm{y}}\,{\rm{o}}{\rm{f}}\,{\rm{b}}{\rm{e}}{\rm{a}}{\rm{d}}{\textstyle \mbox{'}}{\rm{s}}\,{\rm{o}}{\rm{u}}{\rm{t}}{\rm{e}}{\rm{r}}\,{\rm{r}}{\rm{i}}{\rm{n}}{\rm{g}}\,{\rm{i}}{\rm{n}}\,{\rm{g}}{\rm{r}}{\rm{e}}{\rm{e}}{\rm{n}}\,{\rm{c}}{\rm{h}}{\rm{a}}{\rm{n}}{\rm{n}}{\rm{e}}{\rm{l}}$$

$${90}_{or}^{th}=\,{90}^{th}\,{\rm{P}}{\rm{e}}{\rm{r}}{\rm{c}}{\rm{e}}{\rm{n}}{\rm{t}}{\rm{i}}{\rm{l}}{\rm{e}}\,{\rm{p}}{\rm{i}}{\rm{x}}{\rm{e}}{\rm{l}}\,{\rm{i}}{\rm{n}}{\rm{t}}{\rm{e}}{\rm{n}}{\rm{s}}{\rm{i}}{\rm{t}}{\rm{y}}\,{\rm{o}}{\rm{f}}\,{\rm{b}}{\rm{e}}{\rm{a}}{\rm{d}}{\textstyle \mbox{'}}{\rm{s}}\,{\rm{o}}{\rm{u}}{\rm{t}}{\rm{e}}{\rm{r}}\,{\rm{r}}{\rm{i}}{\rm{n}}{\rm{g}}\,{\rm{i}}{\rm{n}}\,{\rm{r}}{\rm{e}}{\rm{d}}\,{\rm{c}}{\rm{h}}{\rm{a}}{\rm{n}}{\rm{n}}{\rm{e}}{\rm{l}}$$

$${M}_{og}={\rm{Maximum}}\,{\rm{pixel}}\,{\rm{intensity}}\,{\rm{of}}\,{\rm{bead}}\mbox{'}{\rm{s}}\,{\rm{outer}}\,{\rm{ring}}\,{\rm{in}}\,{\rm{green}}$$

$${M}_{or}={\rm{Maximum}}\,{\rm{pixel}}\,{\rm{intensity}}\,{\rm{of}}\,{\rm{bead}}\mbox{'}{\rm{s}}\,{\rm{outer}}\,{\rm{ring}}\,{\rm{in}}\,{\rm{red}}$$

By applying these thresholds, we were able to retrieve 30 out of 33 positive beads fed to the sorting device (~91% yield), with 4 false positives and 2 false negatives (~88% accuracy) (Fig. 5c,d).

We performed a detailed statistical analysis aiming to find metrics that would enable sorting of beads from Classes 5 and 6, which exhibit comparable halo and non-uniform fluorescence patterns. Specifically, Class 5 beads are labeled with a red Streptavidin-Albumin conjugate and exhibit non-uniform fluorescence pattern through the bead’s core as well as a partial halo on the corona; Class 6 beads are labeled with a red Streptavidin-Alcohol Dehydrogenase conjugate and exhibit a strong fluorescent halo and low-to-no fluorescence in the bead’s core. Initially, 34 descriptive metrics were extracted from 61 images acquired from Class 5 and 6 beads, and a K-means clustering was performed using the two metrics that showed the largest difference between Class 5 and 6 beads, namely the normalized 99^th^ percentile pixel in the red channel and the normalized 90^th^ percentile pixel of non-bright segment of bead in the red channel (Fig. 5e,f). To extract these metrics, the bead was segmented in regions of high and low brightness. To detect regions of high brightness, a local first order statistic threshold was used with decreased sensitivity toward the bright foreground (in comparison to that used for entire bead detection). Regions of low brightness were detected by subtracting the mask for bright segments from the mask of the whole bead. Based on results of the K-means clustering analysis, a hyperplane was specified to differentiate between the Class 5 and 6 beads. The clusters obtained from unsupervised sorting were compared with the labeled data to assess the accuracy (Number of beads correctly classified/Total number of beads) of the K-means-based clustering. This comparison indicated that these groups can be discerned with a ~87% accuracy, which accounted for the non-homogeneities associated with fluorescence patterns emerging within the samples prepared. There were instances where distinguishing these two classes was not possible even by visual inspection performed by a trained operator.

Collectively, these tests demonstrated the flexible capabilities of this platform in detecting and sorting beads based in various fluorescence intensities and patterns. By discerning populations with either uniform or more complex fluorescence patterns, this system proved fit for automated, unbiased screening of peptide libraries against different protein targets for the identification of synthetic bioactive compounds.

### Screening through a ChemMatrix-peptide library to sort “halo”-like fluorescence pattern

After demonstrating the ability of the proposed device to sort complex halo-type fluorescence patterns, we proceeded with the selection of protein-binding ligands from a library of combinatorial peptides under competitive conditions (target protein mixed with protein impurities). In particular, we focused on the identification of immunoglobulin G (IgG)-binding ligands as a case study. Initially, a one-bead-one-component (OBOC) octameric peptide library X_1_X_2_X_3_X_4_X_5_X_6_X_7_X_8_-GSG was synthesized, wherein X_1_X_2_X_3_X_4_X_5_X_6_X_7_X_8_ represents the variable region and GSG is a glycine-serine-glycine spacer arm. The library was produced on solid phase (ChemMatrix resin) using an equal ratio of alanine, aspartatic acid, tyrosine, arginine, glycine, glutamate, histidine, leucine, glutamine, and serine. The library was then spiked with ~5% in volume of ChemMatrix beads functionalized with the IgG-binding control peptide HWRGWV-GSG. This peptide ligand has been previously shown to selectively bind IgG in complex fluids, including human plasma and Chinese hamster ovary (CHO) cell culture fluids^20,36,37^. The procedures for the synthesis of the library and HWRGWV-GSG on ChemMatrix beads are detailed in the Supplemental Information. The HWRGWVGSG-spiked library was incubated with red-labeled IgG (tagged with Texas Red NHS ester) mixed with green-labeled CHO host cell proteins (HCPs, tagged with Alexa Fluor 488 NHS Ester) and sorted using the proposed device to identify beads with “high” IgG binding and “low” HCP-binding (red only beads). In this context, we aimed to sort and sequence beads displaying selective halo-patterned binding of IgG to verify that the platform is capable of extracting IgG-binding sequences, including HWRGWVGSG. Nonetheless, positive sequences other than HWRGWVGSG were expected due to limited compositional bias in the combinatorial library. Prior to library screening, optimized hyperplanes for sorting positive beads were determined. In order to identify these hyperplanes, ~30–40 positive control beads were flown through the device and quantitative data was extracted. Based on the results acquired by running these beads through the device, the following thresholds were set for a bead to be considered positive: *(i)* α_or_ − α_og_ > 0, *(ii)* α_or_ > 0.25, *(iii)* 90^th^ percentile pixel intensity of entire bead in green channel <0.1, and *(iv)* 95^th^ percentile pixel intensity of entire bead in red channel >0.08. Using the platform, we screened ~200 beads of this library and identified 12 beads as positive. To further verify the existence of fluorescence pattern of interest, the selected positive beads were imaged again individually in a single well. All 12 beads exhibited halo pattern in post-sorting microscopy, which is indicative of platform’s ability in identifying true positive. Finally, the peptides carried by the selected beads were sequenced by Edman degradation^38^ using a Shimadzu PPSQ 33A Protein Sequencer to verify the presence of the control sequence HWRGWV-GSG (Supplemental Figs 3 and 4). Prior to sequencing, the beads were treated at low pH (0.2 M acetate buffer, pH 3.5) and washed to remove all bound proteins. Finally, the peptides were sequenced directly from the collected beads. Nine of the 12 positive beads were sequenced, resulting in 2 beads carrying HWRGWVGSG.

## Conclusions

Screening combinatorial peptide libraries using fluorescence-based readouts is a powerful approach for the identification of protein-binding peptides. With solid-phase libraries, in particular, which feature peptides conjugated on porous beads, fluorescence detection of the beads following capture of the labeled protein target is a successful approach for high-throughput screening of combinatorial solid-phase libraries^18,19^. Despite its success, manual screening is extremely labor-intensive and commercial devices for automated screening are likely unaffordable to academic labs. In this work, we developed a low-cost accessible platform for automated screening of solid-phase peptide libraries that integrates lab-scale microfluidics and microscopy with user-friendly software that enables unsupervised bead imaging and sorting. The device, which can process 100–150 beads per hour, was tested to evaluate yield and accuracy of automated bead sorting. This setup was successfully able to handle beads of various size (~100–300) and flexible enough to detect and sort beads with different fluorescence pattern. To this end, we utilized seven classes of beads featuring different patterns of fluorescence labeling that mimic the appearance of library beads screened against protein targets with different size. The average yield and accuracy of positive beads recovered by the device from mixtures of different classes was found to be 92% and 94% respectively. Particularly encouraging was the recovery of beads with complex fluorescence patterns, which afforded ~88% yield and ~88% accuracy. Notably, the acquisition of the metrics needed to perform the bead sorting was unsupervised; specifically, two bead patterns (*i.e*., non-homogeneous and halo-like) were produced using labeled proteins of different molecular weight, automatically acquired, and successfully utilized to recover positive beads from both classes. This demonstrates that the device provides a cost-efficient, accessible alternative for the automated sorting of bead-based combinatorial libraries with high sensitivity and specificity. As a demonstrative case study, we employed the device for selecting IgG-binding peptides by screening a combinatorial library spiked with IgG-binding HWRGWV-ChemMatrix beads upon incubation with IgG spiked in a complex protein mixture (CHO cell culture fluid). We finally confirmed the presence of IgG-capturing beads by Edman sequencing of the positive beads. This platform achieved high accuracy and yield in sorting beads via incorporation of fluorescence microscopy. In addition, as a microfluidic platform, multiple devices and setups can be assembled in a relatively short period of time.

The proposed device platform can also be integrated with other analytical instruments as well as systems that enhance the decision-making algorithms. Additionally, image processing and pattern recognition can be carried out using machine-learning algorithms that would improve the accuracy of the decisions made during sorting over the currently utilized statistical algorithm. Integrating these supervised machine-learning algorithms would lead to detecting more subtle and complicated patterns occurring throughout screening a library of peptides. The throughput of this platform can also be improved by implementing parallel devices simultaneously or by increasing the concentration of beads in the suspension flown through the device.

## Methods

### Device Fabrication

The microfluidic platform was fabricated by traditional photolithography followed by soft lithography. Negative photoresist SU-8 2150 was spun at 1460 rpm to achieve a feature height of 400 μm (Fig. 1d). Soft bake was carried out for 10 and 90 minutes at 65 °C and 95 °C, respectively. The wafer was then exposed to UV light for 14 seconds using a UV-KUB 3 mask aligner. The mold was then further baked at 65 °C and 95 °C for 5 and 30 minutes respectively to ensure complete cross-linking of exposed regions. Soft lithography was performed in two steps since on-chip valves require a more flexible material for proper operation. First, a thin PDMS layer at a 20:1 ratio of polymer to cross-linker was poured and cured for 20 minutes at 80 °C. A second thick PDMS layer at a 10:1 ratio of polymer to cross-linker was then poured on the mold and cured for 2 hours at 80 °C.

### Preparation of fluorescently labeled beads

Beads were prepared to feature two different fluorescence profiles, namely homogeneous and “halo”-like distributions, as described by Marani *et al*.^18^. Beads with homogeneous fluorescence were prepared by labeling ChemMatrix aminomethyl beads with Texas Red NHS ester, fluorescein isothiocyante (FITC), or both; to this end, 50 mg ChemMatrix dry resin was swollen in 1 ml 0.1 M sodium bicarbonate, pH 8.3, for 1 hour at room temperature and incubated with 50 µl of 2 mg/mL fluorescent dye solution in dimethyl sulfoxide (DMSO) in dark, for 1 hour at room temperature under gentle agitation. After incubation, the dye solution was removed and the beads were washed thoroughly with 0.1% Tween 20 in phosphate buffered saline (PBS), pH 7.4 (PBS-T) for storage and to remove unreacted fluorescent dye. Beads featuring halo-like beads were produced using proteins with a range of molecular weights. To this end, biotin was initially conjugated onto ChemMatrix aminomethyl beads by incubating the resin with a 1 mg/mL solution of biotin and 2 mg/mL 1-Ethyl-3-(3-dimethylaminopropyl)carbodiimide (EDC) in 0.1 M MES, pH 4.6, to achieve a ratio of 1 mg biotin:2 mg EDC:100 mg resin, for 2 hours under gentle agitation at room temperature. Meanwhile, high purity bovine serum albumin (BSA, 66 kDa) and alcohol dehydrogenase (AD, 150 kDa) were dissolved at 2 mg/ml in phosphate buffered saline and conjugated to streptavidin (53 kDa) using a LYNX Rapid Streptavidin conjugation kit. The streptavidin-conjugated proteins were then fluorescently labeled with Texas Red NHS ester. Briefly, 10 µL of streptavidin-conjugated protein was dissolved at 2 mg/ml in 0.1 M sodium bicarbonate, pH 8.3, and mixed with 1 µL of 10 mg/mL solution of Texas Red NHS dye in DMSO under gentle agitation for 1 hour, light-protected at room temperature. The unreacted dye was removed by diafiltration against PBS-T. For labeled protein-bead interactions, 20 µL of the labeled streptavidin and protein conjugates was incubated with 2–5 µl settled volume of biotinylated beads for at least 1 hour at 2–8 °C.

### Experimental setup

In this study, beads are sorted based on their fluorescence intensity or pattern in both red and green channels. Simultaneous dual color fluorescence microscopy was performed using a LEICA DMi8 inverted microscope connected to a Hamamatsu Orca-D^2^ camera equipped with two charge-coupled devices (CCDs) enabling simultaneous microscopy in wavelengths of interest. The ChemMatrix beads used in this study tend to aggregate in solution, leading to clogging of the microfluidic platform and sorting errors (false positives/negatives). To prevent aggregation, beads were maintained in a diluted suspension in PBS buffer (150 beads per 80 ml) and gently stirred on an orbital shaker throughout the duration of the sorting cycle. On-chip valves were filled and degassed with a 50% glycerol solution with a similar refractive index as PDMS, which improves image quality in the vicinity of the valves. A custom-built pressure box equipped with pressure regulators was used to drive fluid flow in the tubing and device. Valve operations was controlled by a custom-developed MATLAB Graphical User Interface (GUI).

### Image processing

To automate the sorting process in an unbiased and quantitative manner, computer-vision algorithms were implemented to detect bead presence in the imaging zone and classify it as negative or positive based on the criteria provided by the operator. Bead detection was carried out by a custom developed MATLAB algorithm that identifies the presence of beads in the channel independently of bead size or fluorescence intensity. As shown in Supplemental Fig. 1, bead detection begins by converting the raw grayscale image to a binarized image using a local first order statistic threshold. The background noise detected in the binarized image is eliminated by removing objects smaller than 100 pixels. The image is then dilated and filled to re-construct the bead structure. To further refine the mask, the image is opened, eroded, and dilated to smooth the bead shape. We integrated this algorithm with the live image acquisition setup where the presence of each incoming bead was detected. Once the bead is detected and isolated in the imaging zone, a second image processing algorithm extracts the image intensity profile using the mask previously generated. Various metrics such as mean intensity, max intensity, intensity N^th^-percentile, and different combinations of these metrics are extracted. The values extracted using this algorithm are normalized by converting the 12-bit pixel value range from 0 (black)-4095(white) to 0 (black)-1 (white). Positive beads are detected and isolated based on the values of these metrics. In this study, more than 20 metrics were extracted for each bead. Depending on the pattern of interest, a combination of two or three metrics is used as criteria for the identification and sorting of positive beads.

## Supplementary information

Supplementary Information

## Data Availability

All images are available upon request.
