# Supplementary material for: Affordable Microfluidic Bead-Sorting Platform for Automated Selection of Porous Particles Functionalized with Bioactive Compounds
Source: Sci Rep. 2019 May 10;9:7210. doi: 10.1038/s41598-019-42869-5 (PMC6510793; doi:10.1038/s41598-019-42869-5)
Supplement: Supplementary file 1 — Supplementary Information [file 41598_2019_42869_MOESM1_ESM.docx]

# **Affordable Microfluidic Bead-Sorting Platform for Automated Selection of Porous Particles Functionalized with Bioactive Compounds**

**Sahand Saberi-Bosari^a^, Mohammad Omary^a^, Ashton Lavoie^a^, Raphael Prodromou^a^, Kevin Day^a^, Stefano Menegatti^a,b,*^, Adriana San-Miguel^a,*^**

^a^ Department of Chemical and Biomolecular, NC State University, Raleigh, NC 27695, USA

^b^ Biomanufacturing Training and Education Center (BTEC), NC State University, Raleigh, NC 27695, USA

* Corresponding authors


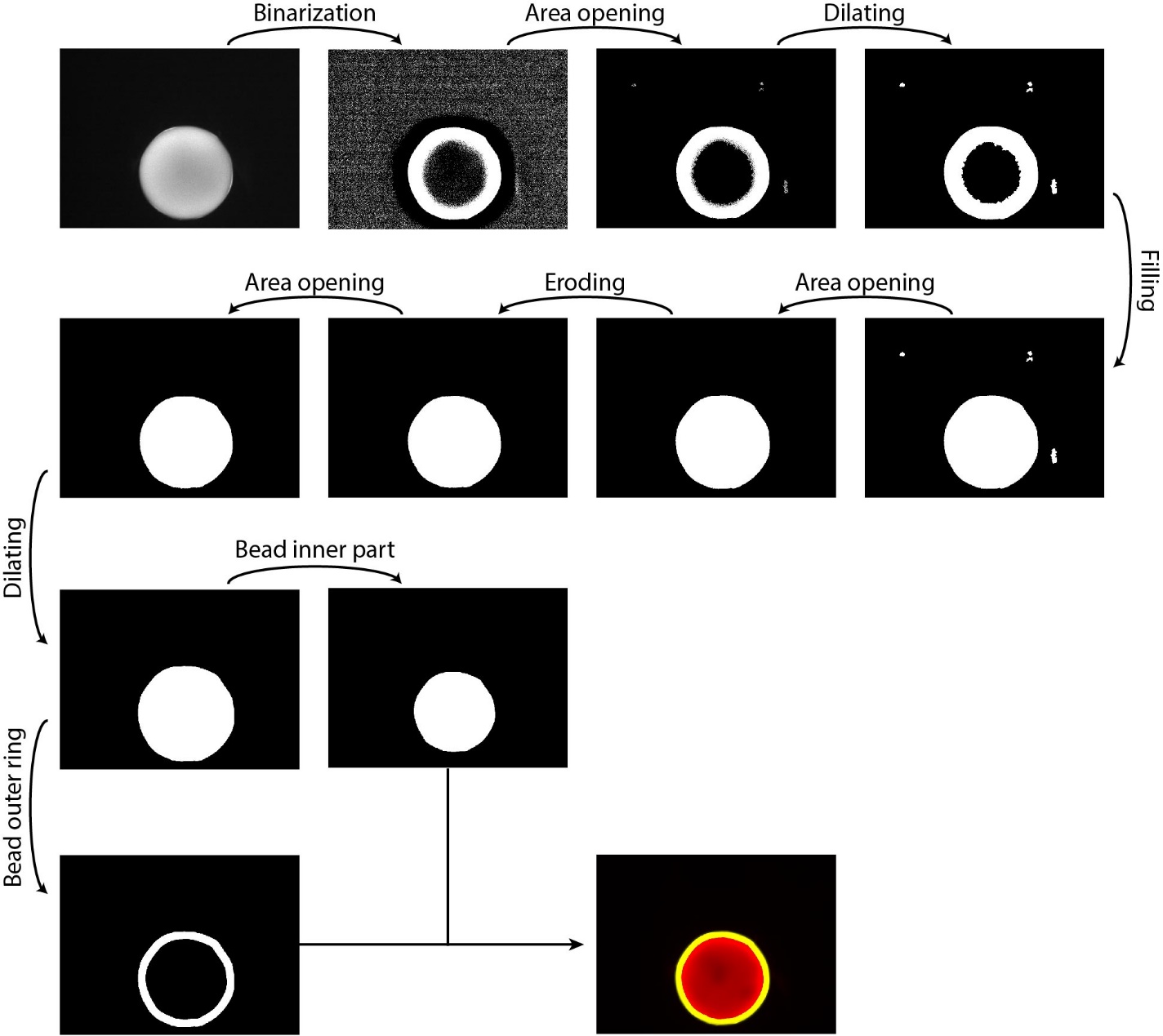


Figure 1 Flowchart of image segmentation.


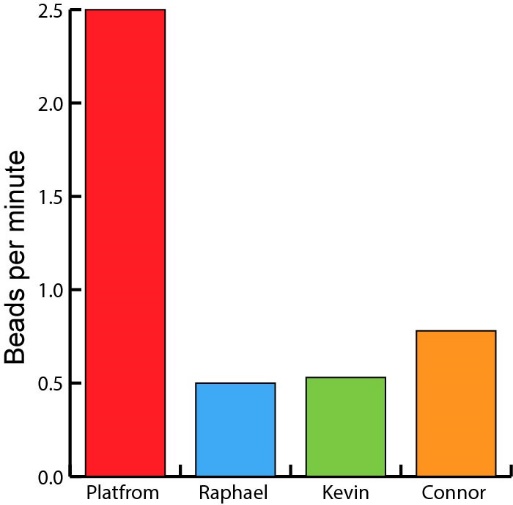


Figure 2 Comparison of speed of bead sorting between trained operators and the microfluidic device. The operators were asked to plate 96 well-plates with single beads and perform fluorescence microscopy of each well.


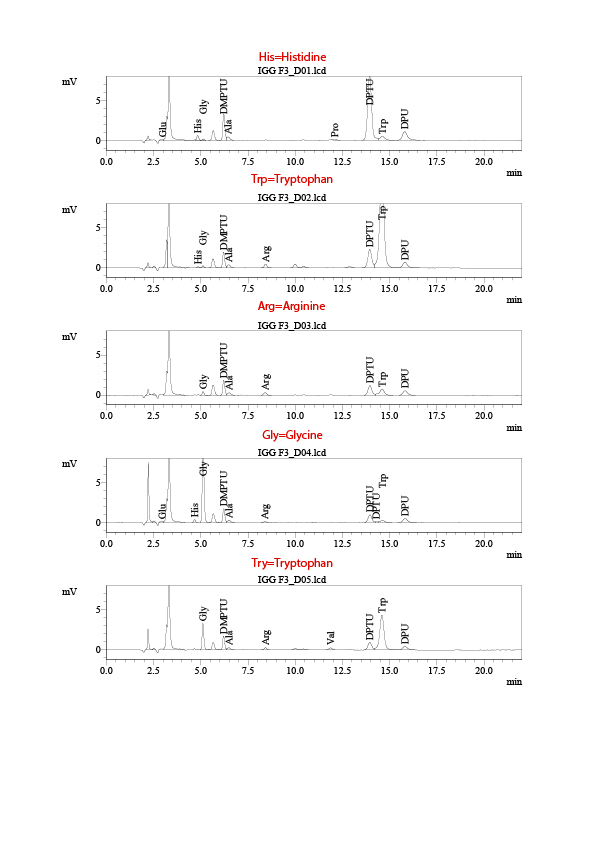

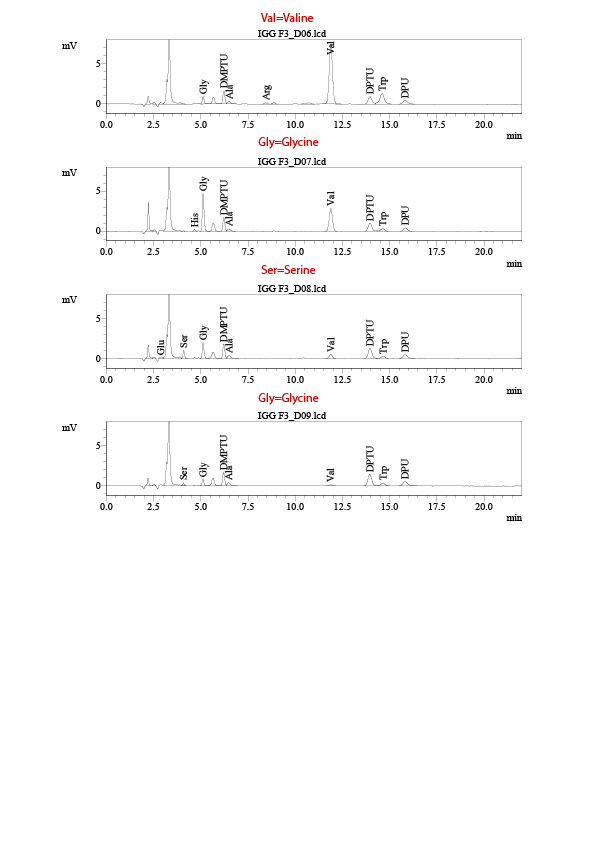


Figure 3 Edman sequencing of positive control bead collected in well F3 from library screening. All amino acid residues were correctly assigned by the Edman data analysis, except for the third and last residues. The Arginine (Arg) peak is the only peak whose intensity does not decrease from the 2^nd^ chromatogram to the 3^rd^ chromatogram, thereby indicating that Arginine is actually located in position 3, as expected. Since the last three amino acids in all sequences of the library and in the spiked IgG-binding peptide HWRGWV-GSG are Gly-Ser-Gly, we can assume that the last amino acid is Glycine.


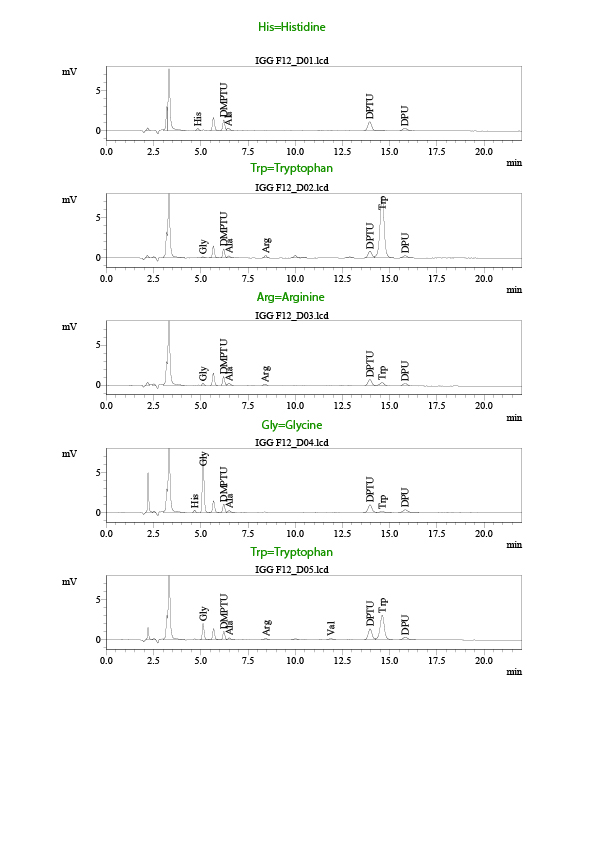

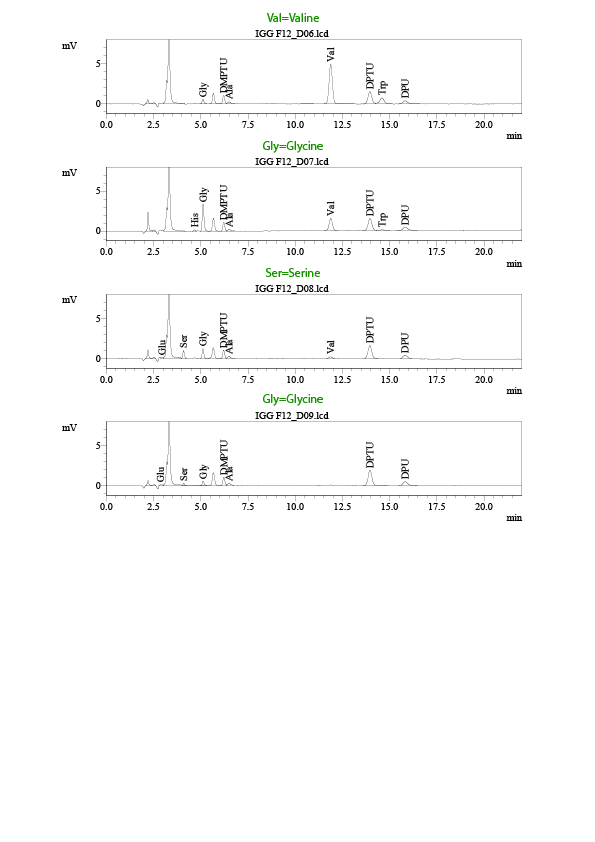


Figure 4 Edman sequencing of a positive control bead collected in well F12 from library screening.

**LIBRARY SYNTHESIS**

The combinatorial octamer peptide X_1_-X_2_-X_3_-X_4_-X_5_-X_6_-X_7_-X_8_ was synthesized by Fmoc/tBu strategy onto 2 g of HMBA-ChemMatrix resin (HMBA: hydroxymethylbenzoic acid) preloaded with the tripeptide GSG (G: Glycine, S: Serine). Ten protected amino acids Fmoc-Ala-OH, Fmoc-Asp(OtBu)-OH, Fmoc-Tyr(tBu)-OH, Fmoc-Arg(Pbf)-OH, Fmoc-Gly-OH, Fmoc-Glu(OtBu)-OH, Fmoc-His(Trt)-OH, Fmoc-Leu-OH, Fmoc-Gln(Trt)-OH, and Fmoc-Ser(tBu)-OH were used as building blocks to produce the combinatorial segment of the library (X_1_-X_8_) via “split-couple-recombine” technique. First, every amino acid was conjugated to a 0.2 g aliquot of resin using 3 equivalents (eq.) of amino acid in DMF, 3 eq. of 2-(7-aza-1H-benzotriazol-1-yl)-1,1,3,3-tetramethyluronium hexafluorophosphate (HATU) in DMF, and 6 eq. of diisopropylethylamine (DIPEA) in NMP at 45˚C for 20 min. After amino acid conjugation and removal of the Fmoc protecting group using 20% piperidine in DMF for 20 minutes at room temperature, the ten aliquots were combined, mixed, and re-divided into 10 aliquots. The steps of amino acid conjugation, Fmoc removal, aliquot mixing, and splitting were repeated seven more times, resulting in a pool of 10^8^ peptide combinations. Finally, the peptides were collectively deprotected using a cleavage cocktail TFA/Thioanisole/EDT/anisole (90/5/3/2) for 2 h. The resulting library was finally washed thoroughly with DMF and stored in 20% aqueous ethanol at 4˚C.

**CONJUGATION OF FLUORESCENT DYES TO PROTEINS**

Human Immunoglobulin G (IgG) and Chinese Hamster Ovary Host Cell Proteins (CHO HCPs) were labelled with Texas Red and Alexa Fluor 488 respectively, following the manufacturer’s protocol. CHO HCPs were dialyzed against 0.1 M Sodium Bicarbonate Buffer pH 8.3 at 10 mg/mL using a Thermo Scientific Pierce 9K MWCO Protein Concentrator. IgG was also dialyzed against 0.1 M Sodium Bicarbonate Buffer pH 8.3 at 10 mg/mL. Texas Red and Alexa Fluor 488 were each dissolved in dry dimethyl sulfoxide (DMSO) at 10 mg/mL and immediately added to the corresponding protein solution at a 1:10 mass ratio of label to protein. The reaction was incubated for 1 hour at room temperature on a rotator, and then diafiltered into 50 mM sodium phosphate, 150 mM sodium chloride, pH 7.4, 0.1% Tween 20 (PBS-T) using Amicon Ultra-0.5 ml 10 kDa MWCO filters to remove any unreacted dye.

**INCUBATION OF BEADS AGAINST IgG and CHO HCP**

To calibrate the instrument with positive control beads, 20 µL of Texas Red-IgG protein in PBS-T was added to 7 µL of settled HWRGWVGSG beads. For the library incubation, 12 µL of settled combinatorial octamer peptide beads were added with 0.5 µL of settled HWRGWVGSG-ChemMatrix beads. Subsequently, 66 µL of 2 mg/mL IgG and 33 µL of 2mg/mL CHO HCP in PBS-T were added to 12.5 µL of the spiked peptide library, achieving a total protein incubation solution of 100 µl of 1.3 mg/mL IgG and 0.7 mg/mL CHO HCP in PBS-T. The positive control and the spiked library were incubated with their respective protein solutions overnight at 4^o^C in the dark. The beads were then washed three times with PBS-T and screened.

**SEQUENCING OF SELECTED BEADS**

After incubation and screening, each selected bead was placed in an individual well of a 96-well plate and washed to remove the bound protein. Specifically, every bead was treated with *(i)* 100 µL of 0.2 M acetate buffer pH 3.5 for three times, at room temperature, for 30 minutes; *(ii)* 100 µL of 50 mM PBS pH 7.4 for three times; *(iii)* copiously rinsed with water and acetonitrile for one minute each. They were then stored in acetonitrile at 4^o^C until sequencing.

On-bead sequencing of selected peptides was conducted via Edman degradation using a PPSQ 33A protein sequencer equipped with an LC-20AT solvent delivery module and an SPD-20A UV-VIS detector (Shimadzu). Following a phenylthiohydanoin (PTH)-amino acid standard, single beads were placed onto a polyvinylidene difluoride (PVDF) membrane and loaded into a PPSQ 33A reactor according to the instrument protocols. The bead is sequenced using the Edman degradation reagents and separated with a Wakopak® Wakosil PTH-II φ4.6mm × 250mm (S-PSQ) column. The sequence was identified by matching amino acid retention times to the calibrated PTH-amino acid standard.
